# What Does ‘Information’ Mean in Integrated Information Theory?

**DOI:** 10.3390/e20120894

**Published:** 2018-11-22

**Authors:** Olimpia Lombardi, Cristian López

**Affiliations:** 1 CONICET–Institute of Philosophy, University of Buenos Aires, Buenos Aires 1406, Argentina; 2 Department of Philosophy, University of Lausanne, CH-1015 Lausanne, Switzerland

**Keywords:** integrated information theory, Shannon information, causation, manipulability theory of causation

## Abstract

Integrated Information Theory (IIT) intends to provide a principled theoretical approach able to characterize consciousness both quantitatively and qualitatively. By starting off identifying the fundamental properties of experience itself, IIT develops a formal framework that relates those properties to the physical substratum of consciousness. One of the central features of ITT is the role that information plays in the theory. On the one hand, one of the self-evident truths about consciousness is that it is *informative*. On the other hand, mechanisms and systems of mechanics can contribute to consciousness only if they specify systems’ *intrinsic information*. In this paper, we will conceptually analyze the notion of information underlying ITT. Following previous work on the matter, we will particularly argue that information within ITT should be understood in the light of a causal-manipulabilist view of information (López and Lombardi 2018), conforming to which information is an entity that must be involved in causal links in order to be precisely defined. Those causal links are brought to light by means of interventionist procedures following Woodward’s and Pearl’s version of the manipulability theories of causation.

## 1. Introduction

In his 1995 paper “Facing up to the problem of consciousness”, David Chalmers introduced the so-called “hard problem of consciousness”, which consists in explaining our inner experiences in physical terms:

The really hard problem of consciousness is the problem of experience. […] It is undeniable that some organisms are subjects of experience. But the question of how it is that these systems are subjects of experience is perplexing. Why is it that when our cognitive systems engage in visual and auditory information-processing, we have visual or auditory experience: the quality of deep blue, the sensation of middle C? How can we explain why there is something it is like to entertain a mental image, or to experience an emotion? It is widely agreed that experience arises from a physical basis, but we have no good explanation of why and how it so arises.

[1] (p. 201)

Since the beginning of the 20th century, this problem was addressed from very different perspectives, both in philosophy and in science. In the field of neurosciences, one of the most promising approaches is that offered by the *Integrated Information Theory* (from now on IIT), proposed by Giulio Tononi and developed by him along with his collaborators. The purpose of IIT is to supply both a qualitative and quantitative approach to consciousness, as well as the relationships with its physical substratum. According to the theory, consciousness (or conscious experience) is characterized in terms of integrated information, that is, information specified by a whole system that cannot be reduced to that specified by its parts.

The explanatory and predictive power of IIT mainly relies on its formal and conceptual accuracy. When applied to specific models, the theory can account for the facts that some simple systems can be minimally conscious, that some complicated systems can be unconscious, and that two different systems can be functionally equivalent, and yet, one is conscious and the other one is not.

This paper will not focus on the technical aspects of IIT. Instead, the purpose here is to conceptually analyze the notion of information underlying IIT, bringing to fore some of its assumptions. Indeed, a theory that proposes a tight link between consciousness and information is committed to take a stand regarding the meaning of ‘information’ within such a framework, thereby adopting one among its several possible interpretations. In particular, we will argue that a manipulabilist view of information is the interpretation that best suits IIT. In this interpretation, information is understood within a causal structure that can be exposed by means of tools provided by manipulability theories of causation (specially, the interventionist version). In order to achieve this purpose, the structure of the paper is as follows. In Section 2, the basics of IIT―in its 3.0 version―will be concisely exposed. In Section 3, the formal difference between integrated information and Shannon information, as presented by Tononi and his collaborators, will be considered. On this basis, in Section 4 we will argue that, by contrast to what is usually assumed in the literature, formal precision in the definition of a concept does not suffice to solve the conundrums stemming from interpretive aspects, which we will illustrate with the case of Shannon information. In Section 5, we will introduce our recently-developed interpretation of information [2], according to which information is an entity that must be involved in causal links in order to be properly defined. In Section 6, the morals drawn from the previous section will be applied to the concept of information underlying IIT. Section 7 will be devoted to show why a manipulabilist notion of information supplies a clear elucidation of the relationship between information and causation in the context of IIT. In Section 8, some criticisms to IIT, related to the concept of information and causation, will be considered. In this section, we will argue that those criticisms are the result of assessing IIT as if it were a philosophical proposal in the context of the philosophy of mind instead of a scientific theory with empirically testable predictions. Finally, the section Concluding Remarks will stress not only our contribution to the elucidation of a central concept in a successful *scientific* theory, but also the advantage of placing IIT in the broader context of the scientific theories that make essential use of the concept of information.

## 2. Some Basics of IIT

Instead of starting from the neural mechanism in the brain to explain how consciousness emerges, IIT starts off by taking the phenomenology of consciousness as primary. Then, it specifies which conditions must be satisfied by physical mechanisms, such as neurons and their connections, so as to explain many known facts about consciousness and its relationships to the brain.

Although IIT has undergone various developments and modifications over the years (see [3,4,5,6]), in this paper we will only focus on the most updated version, *IIT 3.0* [7]. We will put aside the consideration of the differences between IIT 3.0 and the older versions of the theory, because our main interest is the concept of information and its relation with the notion of causation.

ITT 3.0 begins by introducing: (i) five self-evident truth about consciousness, the *axioms* of the theory; and (ii) five necessary ontological assumptions about its physical substratum, the corresponding *postulates*. Briefly, the axioms of the theory are:

- *Existence*: Consciousness exists.
- *Composition*: Consciousness is structured: each experience consists of multiple aspects in various combinations.
- *Information*: Consciousness is informative: each experience differs in its particular way from other possible experiences.
- *Integration*: Consciousness is integrated: each experience is (strongly) irreducible to non-interdependent components.
- *Exclusion*: Consciousness is exclusive: each experience excludes all others.

The postulates of the theory establish the properties that a physical system must instantiate in order to fulfill each of the axioms. A *physical system* (or *system* for short) is a set of elements, each with two or more internal states, with inputs that influence that state, and with outputs that are influenced by that state (for example, neurons or logic gates); the state of the system is defined by the states of its elements. A *mechanism* is a subset of the set of the elements that constitute a system; the state of a mechanism is defined by the states of its belonging elements. Integrated Information Theory 3.0 supplies specific postulates for mechanisms and for systems of mechanisms; here we will only consider mechanisms for they are sufficient for our conceptual purposes. So, the postulates for mechanisms are:

- *Existence*: Mechanisms in a state exist.
- *Composition*: Elementary mechanisms can be combined into higher order ones.
- *Information*: A mechanism contributes to consciousness only if generates information, that is, if it establishes a cause-effect structure that constrains the system’s states that can be its possible causes and effects.
- *Integration*: A mechanism contributes to consciousness only if it generates information (if it establishes a cause-effect structure) that is irreducible to that generated by its independent components.
- *Exclusion*: A mechanism contributes to consciousness at most with one cause-effect structure, the one having the maximum value of integrated information (see below).

The operation of the system is completely specified by the transition probability matrix (TPM), which specifies the probability of transition from each state of the system to any other state. All the relevant magnitudes of the theory can be computed in terms of the TPM. Let us consider the mechanism *M* in its current state *m_c_*, where *M* is included in the system *S*, and certain subsets *Z_t_*_−1_ and *Z_t_*_+1_ of the elements of *S*.

- The *cause repertoire*/the *effect repertoire* of *M* in *m_c_* with respect to *Z_t_*_−1_/*Z_t_*_+1_, $p(Z_{t-1}/m_{c})$/$p(Z_{t+1}/m_{c})$, is the probability distribution over the potential past/future states of the subset *Z_t_*_−1_/*Z_t_*_+1_ as constrained by *M* in *m_c_*.
- The *cause information* $c_{i}(Z_{t-1}/m_{c})$/the *effect information* $e_{i}(Z_{t-1}/m_{c})$ generated by *M* in *m_c_* with respect to *Z_t_*_−1_/*Z_t_*_+1_ is measured as the distance between $p(Z_{t-1}/m_{c})$/$p(Z_{t+1}/m_{c})$ and its unconstrained cause repertoire $p^{uc}(Z_{t-1}/m_{c})$/unconstrained effect repertoire $p^{uc}(Z_{t+1}/m_{c})$, that is, the uniform probability distribution over the subset *Z_t_*_−1_/*Z_t_*_+1_.
- The *cause-effect information* $ce_{i}(Z_{t\pm 1}/m_{c})$ generated by *M* in *m_c_* with respect to *Z_t_*_−1_ and *Z_t_*_+1_ is computed as the minimum of $c_{i}(Z_{t-1}/m_{c})$ and $e_{i}(Z_{t-1}/m_{c})$.
- In all the cases, the distance between probability distributions is evaluated through the so-called *Earth mover’s distance*, which quantifies how much two distributions differ from one another, but taking into account the distance between states of the system.

Intuitively, given a system *S*, if its mechanism *M* in its current state *m_c_* specifies nothing about the past states of *Z_t_*_−1_, the cause repertoire of *M* in *m_c_* is identical to its unconstrained distribution: *M* in *m_c_* is completely unselective with respect to *Z_t_*_−1_, that is, it generates no cause information $c_{i}(Z_{t-1}/m_{c})$. By contrast, if the mechanism *M* in its current state *m_c_* specifies univocally the past states of *Z_t_*_−1_, it is maximally selective with respect to *Z_t_*_−1_: the cause information $c_{i}(Z_{t-1}/m_{c})$ generated by *M* in *m_c_* is maximal. In a nutshell, the more selective a mechanism in its current state is, the more information it generates.

However, IIT 3.0 is not uniquely interested in the information generated by parts of the mechanism, but what really matters for consciousness is the *integrated information*, that is, the information generated by the whole mechanism above and beyond the information generated by its parts when considered as causally independent. According to the *Integration Postulate*, only mechanisms that specify integrated information are capable of contributing to consciousness. In this sense, mechanisms are informationally irreducible. The central notions as for integrated information, mechanisms, partitions and their relationships are as follows:

- The *partition* $P_{t\pm 1}=\left\{P_{t-1},P_{t+1}\right\}$ is a pair of partitions of the sets *Z_t_*_−1_ and *Z_t_*_+1_, such that $P_{t-1}=\left\{Z_{t-1}^{1},Z_{t-1}^{2}\right\}$ and $P_{t+1}=\left\{Z_{t+1}^{1},Z_{t+1}^{2}\right\}$.
- The *cause*/*effect integrated information*, $\varphi_{\mathrm{cause}}(m_{c},Z_{t-1},P_{t-1})$/$\varphi_{\mathrm{effect}}(m_{c},Z_{t+1},P_{t+1})$, generated by a mechanism *M* in its current state *m_c_* with respect to the partition /$P_{t+1}$ of the sets *Z_t_*_−1_/*Z_t_*_+1_ is computed as the distance between the cause repertoire/the effect repertoire of *M* in *m_c_*, $p(Z_{t-1}/m_{c})$/$p(Z_{t+1}/m_{c})$, and the cause repertoire/the effect repertoire of *M* in the state *m_c_* with respect to the partition $P_{t-1}$/$P_{t+1}$, $p(Z_{t-1}^{1}/m_{-c}^{1})\times p(Z_{t-1}^{2}/m_{-c}^{2})$/$p(Z_{t+1}^{1}/m_{+c}^{1})\times p(Z_{t+1}^{2}/m_{+c}^{2})$, where $m_{-c}^{1}$, $m_{-c}^{2}$/$m_{-c}^{1}$, $m_{-c}^{2}$ are the states of the sub-mechanisms corresponding to the partition $P_{t-1}$/$P_{t+1}$.
- The *integrated information* $\varphi (m_{c},Z_{t\pm 1},P_{t\pm 1})$ generated by a mechanism *M* in its current state *m_c_*, with respect to the partition $P_{t\pm 1}$ of the sets $Z_{t\pm 1}$ of the system’s states is computed as the minimum of $\varphi_{\mathrm{cause}}(m_{c},Z_{t-1},P_{t-1})$ and $\varphi_{\mathrm{effect}}(m_{c},Z_{t+1},P_{t+1})$.
- But a system can be partitioned into many ways. In the context of IIT 3.0, the relevant partition is the *Minimum Information Partition*.
  The *minimum information partition*, MIP, is the partition corresponding to minimum integrated information: $\varphi^{\mathrm{MIP}}(m_{c},Z_{t\pm 1})=\min_{P_{t\pm 1}}\left(\varphi (m_{c},Z_{t\pm 1},P_{t\pm 1})\right)$.
- The *integrated information* $\varphi (m_{c},Z_{t\pm 1})$ generated by a mechanism *M* in its current state *m_c_* with respect to the sets $Z_{t\pm 1}$ is $\varphi^{\mathrm{MIP}}(m_{c},Z_{t\pm 1})$.

Up to this point, generic subsets *Z_t_*_−1_ and *Z_t_*_+1_ of the elements of *S* were considered. These subsets define a cause-effect repertoire which, in turn, defines the *integrated information* $\varphi (m_{c},Z_{t\pm 1})$ of the mechanism *M* in its state $m_{c}$. But the *Exclusion Postulate* establishes that a mechanism contributes to consciousness at most with one cause-effect structure. Integrated Information Theory 3.0 selects the effective cause-effect repertoire that corresponds to maximum integrated information:

- The *maximally irreducible cause-effect repertoire*, MICE, is the cause-effect repertoire corresponding to a maximum integrated information among all possible subsets $Z_{t\pm 1}$:$\varphi^{\mathrm{MICE}}(m_{c})=\max_{Z_{t\pm 1}}\left(\varphi (m_{c},Z_{t\pm 1})\right)$.
- The *integrated information* $\varphi (m_{c})$ generated by a mechanism *M* in its current state *m_c_* is $\varphi^{\mathrm{MICE}}(m_{c})$.

By transferring the concept of integrated information φ from the level of mechanisms to the level of a *system of mechanisms*, IIT 3.0 offers the definition of the integrated conceptual information (“big phi” Φ, as opposed to “small phi” φ at the level of mechanisms). Precisely, the *integrated conceptual information* Φ is the conceptual information that is generated by a system above and beyond the conceptual information generated by its minimal parts, where *conceptual information* is the analog at the system level of cause-effect information at the level of mechanisms (see details in [7]).

Notice that, since integrated conceptual information is a mark of consciousness, then consciousness comes in degrees corresponding to Φ values. This means that IIT, since its first versions, implies a form of panpsychism. In fact, even a simple system such a single photodiode is conscious to some degree, if it is not contained within a larger complex: “even a binary photodiode is not completely unconscious, but rather enjoys exactly 1 bit of consciousness” [4] (p. 236). In a similar sense, “qualia” can be attributed to simple logical gates [6]. What distinguishes human consciousness from the low-level consciousness of simple systems is its richness and massive integration. The measure of integrated conceptual information is designed precisely to represent those systemic features.

## 3. Differences between IIT Information and Shannon Information

When the notion of information appears in scientific theories with a relevant theoretical role, Shannon information immediately enters the scene. This is reasonable because Shannon’s theory was the first in providing a formally accurate definition of information, whose usefulness rapidly exceeded the boundaries of the field in which it was proposed. Perhaps for this reason, it is sometimes assumed that any reference to information needs to comply with Shannon theory. This seems to be implied in some criticisms held against IIT. For instance, John Searle [8] claims that Tononi and his collaborators want consciousness to be measurable using the mathematics of Shannon’s information theory. The same idea underlies Michael Cerullo’s criticisms against IIT: “A purely data-defined theory of information such as Shannon’s lacks the ability to link information with the causal properties of the brain […] Only by including syntactic, and most importantly semantic, concepts can a theory of information hope to model the causal properties of the brain.” [9] (p. 58). Nevertheless, although the concept of information is central to IIT, Tononi and his collaborators stress that IIT’s notion of information is clearly different from that of Shannon theory. The examination of this claim is the first step toward the elucidation of the notion of information underlying IIT.

To begin with, it is quite clear that Shannon theory’s formalism and IIT’s formalism are different. Moreover, as Tononi and his collaborators themselves claim, IIT and Shannon theory diverge from one another with respect to the meaning of ‘information’. In Shannon theory, information is transmitted from a source to a destination, where the source and the destination are systems precisely characterized in terms of their possible states with their corresponding probabilities. The information of a system is computed in terms of the probability distribution over its states, as the weighted average of the negative logarithms of the probabilities of the states. In this context, the relevant probabilistic relations between two systems are encoded in the mutual information, which is computed in terms of the probability distributions of the states of the two systems and the conditional probabilities of the states of one system given the states of the other. As repeatedly stressed, semantic content plays no role in Shannon theory:

Frequently the messages have *meaning*; that is, they refer to or are correlated according to some system with certain physical or conceptual entities. These semantic aspects of communication are irrelevant to the engineering problem.

[10] (p. 379; italics in the original)

By contrast, IIT claims that “information *is* meaning” [7] (Text S3, p. 1). This claim is based on the fact that each whole mechanism, with its maximally irreducible cause–effect repertoire and its corresponding integrated information, is conceived as a *concept*, whose meaning expresses the causal role of the mechanism within the system. On this basis, the meaning of each individual concept in the system is self-generated, self-referential, and holistic: it is constructed by the elements of the system, over the elements of the system, and within the context provided by other concepts in the same system. This meaning of ‘meaning’ is completely absent in Shannon theory. However, it is not this self-generated, self-referential, and holistic notion of “meaning” what Shannon mentions in his quote: in the traditional semantic sense, the meaning of a word (or concept) has nothing to do with causation; it is constituted, at least partially, by the extra-linguistic entity the word refers to.

Another relevant difference between the two notions of information is, according to the authors, that, whereas IIT information is intrinsic, Shannon information is extrinsic. Integrated Information Theory information is intrinsic for it is assessed from the intrinsic perspective of a system in terms of the differences that make a difference to it. By contrast, Shannon information is always defined from the extrinsic perspective of an observer describing the statistical dependence between inputs and outputs. This distinction is closely related to the different role that, it is argued, causation plays in the two theoretical contexts. Integrated Information Theory information is causal, and it must be evaluated not just by observing a set of elements, but by perturbing them with the purpose of identifying the causal links in which they are involved. Shannon information is rather characterized by IIT’s advocates as based on the statistical dependence between the inputs and the outputs of a system, independently of the mechanisms that link those inputs and outputs. This distinction between IIT information and Shannon information presupposes an interpretation of Shannon information that makes it a mere measure of statistical correlations.

This way of characterizing the differences between IIT and Shannon theory manifests not only some features about the notion of information Tononi and collaborators endow their own theory with, but also how they conceive Shannon information. It seems that, given the mathematical precision of Shannon theory, the interpretation of the concept of Shannon information is univocally and completely determined. However, this is not quite so. As it will be shown in the next section, when the notion of information is considered from a conceptual angle, many debates about the interpretation of Shannon information are still alive. The review of these debates will offer us the conceptual tools to examine and to sharpen the interpretation of the concept of information in the framework of IIT.

## 4. The Interpretation of Shannon Information

Shannon theory’s formalism certainly constrains the possible interpretations of the terms included in it, but this does not mean that it univocally fixes a single interpretation. In particular, nothing in the formalism tells us what the term ‘information’ refers to within the context of the theory. This fact has led to very different interpretations of the notion of information, even among those who completely agree on the formal aspects of Shannon theory [11,12,13]. At least three interpretations have been proposed so far: epistemic, physical, and syntactical.

According to the epistemic interpretation, information is something that provides knowledge that modifies the state of knowledge of those who receive it. For those that distinguish between an everyday notion of information and a technical one, the link between information and knowledge is only a feature of the everyday notion of information, but not of the technical concept (e.g., [14]). However, that link can frequently be found in technical literature, both in philosophy and in science. For instance, in the field of philosophy, Fred Dretske considers that: “information is a commodity that, given the right recipient, is capable of yielding knowledge.” [15] (p. 47). Also, in sciences the concept of knowledge is placed at the core of the concept of information; along the same line, Jon M. Dunn defines information as “what is left of knowledge when one takes away belief, justification and truth” [16] (p. 423). In the field of physics, Anton Zeilinger even equates information and knowledge when he says that “[w]e have knowledge, i.e., information, of an object only through observation” [17] (p. 633). In the quantum context, out of Shannon theory’s framework, Christopher Fuchs, in adhering to a Bayesian interpretation of probability, advocates for an epistemic interpretation of information [18].

Although from the epistemic perspective information is not a physical item, in general it is assumed that the possibility of acquiring knowledge about the source of information by reading the state of the destination is rooted in the lawfulness of the regularities connecting source and destination. This means that the conditional probabilities necessary to compute the relevant magnitudes of Shannon theory must be determined by natural laws; if those conditional probabilities are represented accidentally, merely by de facto correlations, the states at the destination would tell us nothing about what happened at the source.

A serious problem for the epistemic view concerns the relationship between information and communication. Let us consider a TV transmitter *T* that broadcasts a signal, which is received by two TV sets *A* and *B*: although there is no physical interaction between the two TV sets, the correlations between their states are not accidental, but result from the physical dependence of those states on the states of *T*. Therefore, from an epistemic interpretation, nothing prevents us from admitting the existence of an informational link between the two TV sets. In fact, we can define a communication channel between *A* and *B* because it is possible to learn something about *B* by looking at *A* and vice versa:

From a theoretical point of view […] the communication channel may be thought of as simply the set of depending relations between [a system] *S* and [a system] *R*. If the statistical relations defining equivocation and noise between *S* and *R* are appropriate, then there is a channel between these two points, and information passes between them, even if there is no direct physical link joining *S* with *R*.

[15] (p. 38)

A promissory way out would be to show that a causal structure underlies communication. That implies that in a communicational context some agent may perform a modification at some place that will have consequences somewhere else. This allows agents to *control* a desired effect by acting upon the cause. Evidently, in the case of the two TV sets, no control is possible: nothing can be done, say, at the *A* end that will affect what happens in the *B* end. In other words, the change of the state of *A* cannot be used to control the state of *B*; so, something of the usual conception of communication is missing.

For the physical interpretation, the tenet is that “[i]nformation is physical” [19] (p. 23). This view is based on the idea expressed by the well-known *dictum* “no information without representation”: the transmission of information between two points of space necessarily requires an information-bearing signal, that is, a physical process propagating from one point to the other. Rolf Landauer explicitly advocates for this position when he claims that:

[i]nformation is not a disembodied abstract entity; it is always tied to a physical representation. It is represented by engraving on a stone tablet, a spin, a charge, a hole in a punched card, a mark on a paper, or some other equivalent.

[20] (p. 188), see also [19]

This interpretation has also been supported in the philosophy of science; for instance, Peter Kosso states that “information is transferred between states through interaction.” [21] (p. 37).

The physical interpretation can of course admit some degrees. For the strongest version of the physical interpretation, information is a *substance*: information is a new kind of entity in its own right, something out there in the world. Its essential feature consists in its capability of “flowing” through the physical space, that is, of being generated at one point and transmitted to another point; it can also be accumulated, stored and converted from one form to another. However, according to a weaker version, information is a *property* of the carrier signal; by the term ‘information’ we are not referring to new stuff, but to a property that physical entities may instantiate (for instance, carrier signals or sources). Therefore, even if properties do not flow, the picture of the “flow” of information still makes some sense: the propagation of a physical signal always links transmitter and receiver, and information is a property of such signal.

The physical interpretation is the commonest in the fields of physics and engineering, where the link with knowledge is not relevant: the transmission of information can be used only for control purposes, such as operating a device at the destination end by modifying the state of the source. Furthermore, the need of a carrier signal seems appropriate as physical influences are usually understood through physical interactions. In this light, information is commonly compared to energy, which entered the domain of physics as a mere tool to describe what we can do with physical systems—to perform work—but gradually became an essential item that plays a central unifying role in physics: energy is an item essentially present in all contemporary physical theories. In the light of the strong presence of the concept of information in present-day physics, several authors ([22,23]; Rovelli, personal communication) consider that it is following a historical trajectory analogous to that followed by the concept of energy in the nineteenth century.

Despite its wide acceptance within the physics and engineering community, the physical interpretation had to face a very serious challenge when quantum mechanics came into play with the phenomenon of entanglement. In the quantum scenario, the aim of communication is the same as in the classical case: to identify the message produced at the source by means of the message received at the destination. So the nature of information is the same as in classical communication: there is no “quantum information” qualitatively different from classical information; it refers to information when it is encoded in quantum systems [18,24] (see discussion in [25]). Nevertheless, in the quantum case, entanglement-assisted communication opens the door to transmission of information without physical carrier.

In teleportation, the typical case of entanglement-assisted communication, an unknown quantum state is transferred between two points of space with the assistance of a shared pair of particles prepared in an entangled state, and of two classical bits sent through a classical channel. Although the two classical bits are transferred by means of a physical carrier, there is not physical signal carrying the teleported state. From the physical interpretation’s viewpoint, this is quite perplexing. In order to overcome such perplexity some physicists have devised some unnaturally extreme solutions to find a physical link between the two points, which would be responsible for transporting the quantum state (see discussion in [25]). For instance, some have considered that information travels backwards in time until the event at which the entangled pair was produced, and then travels forward to the future until the time in which the teleported state is recovered [26,27,28]. Others have supposed that the information involved in the quantum state is carried by the classical bits, somehow hidden in the classical signal [29].

Despite the fact that the physical interpretation has been the most usual in the traditional textbooks for training engineers, this situation has changed lately, and recent textbooks overall explain information theory in a formal way, with no mention of sources, receivers or signals. The basic concepts are instead introduced in terms of random variables, probability distributions over their possible values and correlations between them (see, e.g., [30]). This trend can be viewed as embodying a formal interpretation, according to which the term ‘information’ does not refer to something that exists out there in the world in any sense, but neither does it have to do with knowledge. Strictly speaking, the word belongs not to empirical science but to formal science: it has no extra-linguistic reference in itself but its “meaning” has only a syntactic dimension:

[O]nce it is recognized that ‘information’ is an abstract noun, then it is clear that there is no further question to be answered regarding how information is transmitted in teleportation that goes beyond providing a description of the physical processes involved in achieving the aim of the protocol. That is all that ‘How is the information transmitted?’ can intelligibly mean; for there is not a question of information being a substance or entity that is transported, nor of ‘the information’ being a referring term.

[31] (p. 599)

Hence, information is a purely formal concept and the theory of information is said to be a chapter of the theory of probability (see, e.g., [32,33]).

This syntactic (or formal) interpretation is free from the difficulties that challenge the physical interpretation: if the word ‘information’ has no reference to something in the world, the problems of explaining how information is transferred in teleportation suddenly disappear. Notwithstanding this advantage, conceiving of information as a merely formal concept leads to a problem even more serious than that faced by the epistemic interpretation: the magnitudes involved in the mathematical theory of information lose even their lawful ingredient. For instance, the mutual information between two random variables can be defined even if there is no lawful relationship between them and their conditional probabilities expressing only de facto correlations. Therefore, the formal interpretation not only lacks the element of *production* implicit in communication, but furthermore deprives information from his capability of supplying knowledge.

## 5. A Manipulabilist View of Information

As explained in the previous section, despite the precision of the formalism, the concept of information in science has received several interpretations, none of them free from difficulties. On the one hand, although empirical sciences would need a clear concept of physical information, the physical interpretation is seriously challenged by cases of communication appealing to quantum resources. These cases are not exotic, but rather common in present-day engineering: much technology-oriented research has lately gravitated around them. On the other hand, the epistemic and the syntactic interpretations are based on the mere statistical correlations between source and destination; but this strategy is too broad, because it allows characterizing as communicational cases that clearly do not involve transmission of information. Therefore, the challenge is to retain a minimal physical interpretation of information not requiring a physical carrier for transmission. The clue perhaps lies in a basic and intuitive idea: the intuition that a change in the state of the source produces, *causes* a change in the destination. However, the concept of causation is a hard nut to crack. As of the very dawn of philosophy, causation was a puzzling notion, and many diverging conceptions were developed along the centuries. Thus, if the concept of causation is not properly elucidated, one risks replacing a bewildering concept by one equally or even more confusing. It is then necessary to establish how causation is understood or, at least, to supply a criterion to identify causal links.

A wide panoply of approaches to causation can be found in the literature, such as regularity theories (inspired by Hume [34]), counterfactual theories [35], universals-based theories [36], and physical theories [37]. Even the idea of completely wiping out the word ‘cause’ from our scientific vocabulary (as famously proposed by Bertrand Russell [38]) has had its advocates. From a physical perspective, then, causation has been conceived in terms of energy flow [37,39], of *physical processes* [40,41], and of *property transference* [42,43]. However, all of the physical views involve physical signals or space-time connections and, as a consequence, they do not provide the kind of concept of causation necessary for a theory that remains completely agnostic about the nature of causal links. In a recent paper [2], we have proposed a manipulabilist interpretation of information, based on the manipulabilist view of causation.

Regardless of the many highly-sophisticated theories of causation, people act day-to-day steered by the tacit belief that there are actual causal relationships in the world. Most of us are able to identify direct causal relationships, and also to distinguish them from correlations that are merely accidental or due to common causes—anyone can easily distinguish the pain of a finger due to a hammer blow from the showing up of the paperboy when the sun comes up every morning. Similarly, in science, a chemist distinguishes the kind of action that a catalyst performs in increasing the rate of a reaction from the mere correlation between the melting point and the color of an element. But what is this intuitive distinction grounded in?

One of the most basic features of causation is that it makes it possible to exert some control on, or to manipulate, the alleged effect. Nancy Cartwright [44] emphasizes this feature by noting that causal relationships are needed to ground the distinction between *effective* and *ineffective* strategies: an effective strategy proceeds by intervening on a system in order to obtain a desired outcome, and this is possible if and only if a causal link exists. To put it differently, not mere correlations but only causal relationships are exploitable to bring about a certain outcome [45].

The *manipulability accounts of causation* were designed to capture this intuitive feature of causation. The basic idea is that causal relationships are those “potentially exploitable for purposes of manipulation and control” [46] (p. 25). Early versions of the manipulability theories [47], as well as some recent versions advocated by Huw Price and Peter Menzies [48,49], appeal to the notion of free-agency in order to support a reductive approach to causation. But as a consequence of the many criticisms that the free-agency theories received (based on circularity and anthropocentrism, see [50] for details), a non-reductionist version of the manipulability view has been then proposed. Mainly supported by James Woodward [46,51] and Judea Pearl [52], the interventionist version begins by assuming that causation is a primitive notion that cannot be reduced to simpler concepts. On this basis, the purpose of the interventionist version is not to offer a metaphysical elucidation of causation, nor to define causation in terms of non-causal notions, but rather to offer an overarching criterion capable of delimitating the domain of causation through the possibility of exerting control and manipulation within a given system.

What is a causal relationship? What does ‘*X* causes *Y*’ mean? From the manipulability view, ‘*X* causes *Y*’ means, roughly, that we can manipulate *Y* (the *effect*) by manipulating *X* (the *cause*). That is, we can carry out a modification on *Y* by intervening on *X*, and this allows us to control *Y*. Quoting Woodward:

The claim that *X* causes *Y* means that, for at least some individuals, there is a possible manipulation of some value of *X* that they possess, which, given other appropriate conditions (perhaps including manipulations that fix other variables distinct from *X* at certain values) will change the value of *Y* or the probability distribution of *Y* for those individuals.

[46] (p.40)

Mathias Frisch clearly expresses the same idea: “the results of interventions into a system are a guide to the causal structure exhibited by the system” [45] (p. 78).

It is worth emphasizing that this manipulabilist view does not turn causation into an extrinsic phenomenon. The fact that an intervention can be applied from the outside of the system does not imply that the causal link is external: the causally external intervention reveals an intrinsic causal relationship, the causal link one is interested in. Specifically, manipulability manifested through external interventions is only a tool to reveal internal causal links *within* a system.

The main aim of this interventionist version is to supply a clear *operational* criterion for causation, something particularly useful in the empirical sciences. Philosophers can and must discuss about the necessary and sufficient conditions to correctly apply the word “cause” in different domains, even in very atypical and borderline cases. But in science one needs an unequivocal criterion to decide, for instance, if heating a gas causes its expansion, or if the triggering of neuron *A* causes the triggering of neuron *B*. The manipulabilist account of causation would indeed offer such a criterion.

When this idea is used in the framework of the physics of information, then the transmission of information turns out to be rooted in the causal structure of the communicational arrangement, which can be revealed by manipulabilist interventions [2]. Therefore, correlations that are merely accidental or due to a common cause, although introducing a mapping from source to destination, are not enough for transmitting information: for informational links to be adequately defined, an underlying causal structure is strictly required, understood in terms of the possibility of controlling or producing a change in the destination by performing an intervention on the source.

Let us recall the case of the TV transmitter *T* that broadcasts a signal received by two TV sets *A* and *B*. By intervening on *T*, by switching it off for example, the state of the TV set *A* changes, say, from on to off; therefore, there is a causal link between *T* and *A*, which was brought to light by the intervention. Alternatively, no matter which intervention one carries out upon *A*, no modification will result in *B*, and this fact is the manifestation of the causal independence between *A* and *B*. In the case of teleportation, by contrast, communication requires not only the quantum state to be transferred, but also the two classical bits. If the intervention on the source changes the state to be teleported, then the state received also changes. If the intervention is set to block one of the classical bits to be sent through the classical channel, then the teleported state cannot be recovered at the destination end. It is worth stressing that the consequences of the interventions are well known on the basis of experimentally grounded reasons, independently of any interpretation of quantum mechanics

It is worth noticing that our approach is not the first that links information and causation: some philosophers have thought of causation as transmission of information. The idea was probably first introduced by John Collier [53] in explicit terms, who sought to conceive causation fundamentally as an information–transmission process. In Collier’s own words: “the basic idea is that causation is the transfer of a particular token of a quantity of information from one state of a system to another” [53] (p. 215), see also [54]. However, it is important to stress that we are not taking that direction of the elucidatory arrow. We do not want to elucidate causation in terms of information, but the other way around: information in terms of causation. To our knowledge, the road has not been traveled yet in this opposite direction. Thus, in order to avoid misunderstandings, we need to make perfectly clear what our proposal does not intend to render. Firstly, (a) we will not argue that information *is* causation in some fundamental sense and, secondly, (b) at the end of the day, we will remain neutral with respect to what causation *ontologically* is. No doubt ontological matters are greatly interesting, but they are not our concern in this context: our only aim here is to provide a strong and reliable criterion that allows us to bare the causal structure underlying the transmission of information.

In the next section we will see how the above discussion on the interpretation of information may contribute to give an account for the concept in the context of IIT 3.0.

## 6. Interpreting IIT Information

Some scientists, in particular physicists, believe that an empirical theory, when formulated formally, univocally fixes its own adequate interpretation. Logicians, on the other hand, warn that such a belief is blatantly wrong: any formalism admits many potentially infinite interpretations; in particular, many interpretations make the theory true. To put it slightly differently, an empirical theory is a formalism plus an interpretation that endows the terms of the theory with an extra-linguistic meaning.

The remark above fairly applies to IIT: it is not a formal theory, but an empirical theory *about consciousness*, which happens to be based on a precise formalism and is committed to supply an interpretation for its formal terms. In particular, the term ‘information’ must be endowed with a specific meaning in the context of IIT. The discussion of the previous section will help us to elucidate the relevant concept of information in this case.

On the one hand, since IIT is an empirical theory that strongly links consciousness with integrated information, information cannot be conceived as a mere syntactic notion, blind to the lawful relations between the correlated magnitudes: in IIT, causation is what expresses the laws of nature supporting the informational structure of consciousness. In other words, a scientific theory about consciousness that would turn it into a merely syntactic item would not make sense. On the other hand, IIT is a scientific theory about consciousness, conceived as an objective phenomenon of the natural world. Therefore, to the extent that integrated information is what accounts for consciousness, information must be objective in some respect. Hence, the term ‘information’ cannot merely refer to subjects’ knowledge: IIT intends to describe not scientists’ beliefs, but a phenomenon that in some sense exists independently of one’s knowledge. This fact, thus, blocks the epistemic interpretation of the concept of information in the framework of IIT: integrated information is an objective feature of the studied system, which does not depend on the epistemic access of observers.

The physical interpretation of information might be supposed to be the appropriate one in the context of IIT, due to the very aim of the theory: to understand how consciousness is related to its physical substratum. Integrated information, which accounts for consciousness, would then belong to that physical substratum. However, this conclusion has come too quick. When the nature of information is taken into account, such a conclusion can no longer be obtained so straightforward.

As previously remarked, the new protocols of communication show that, by contrast to the traditional assumption, a carrier signal is not necessary for the transmission of information. This fact is what poses a strong challenge to the physical interpretation of information, which requires a physical carrier transmission. Consistently, there is nothing in IIT requiring a signal to establish the links among the elements of a system. Integrated Information Theory introduces no constraint into the physical way in which the elements influence each other: not the physical properties but only the structural features of those links are relevant for the emergence of consciousness.

Summing up, the concept of information underlying IIT 3.0 does not easily fit in the traditional interpretations discussed in the field of the philosophy of information. In this framework, information is not an epistemic or syntactic item, but an objective item that accounts for the objective phenomenon of consciousness. Nevertheless, the concept of information cannot be endowed with the traditional physical interpretation to the extent that it is not tied to the existence of a physical signal that carries information through space. Despite this, it is clear that within IIT causation plays an essential role in the account of consciousness and is closely tied to the concept of information. Therefore, our manipulabilist interpretation of information, which, as argued, supplies a satisfactory explanation of the informational links (both in intuitively simple cases and in more puzzling situations), seems particularly adequate also in the case of IIT.

Relevantly, the manipulabilist view is strongly aligned with the use of ‘information’ in the IIT 3.0 framework. First, information is already closely related to causation within IIT:

A mechanism in a state generates information only if it constrains the states of a system that can be its possible causes and effects—its *cause-effect repertoire*.

[7] (p. 3)

Second, although IIT aims at understanding the relation between consciousness and its physical substratum, the theory does not rely on any particular physical theory to characterize causation, and consequently information. Rather, IIT appeals to the idea of “differences that make differences”:

A mechanism can contribute to consciousness only if it specifies «differences that make a difference» within a system.

[7] (p. 3)

But since the contribution of a mechanism to consciousness is given by its generation of information as causally conceived, it is clear that causation itself is interpreted in terms of “differences that make differences”. This may in turn be straightforwardly translated into manipulabilist terms: a causal relation can be said to hold, if a difference is made in the effect by making a difference in the case by means of an intervention.

This manipulabilist view of causation is not only expressed as a generic view, but also plays a relevant and well-defined role in the *formalism* of IIT, as it dictates how the relevant magnitudes of the theory must be computed. In fact,

Intrinsic information is causal, and it must be evaluated by perturbing a set of elements in all possible ways, not just by observing them.

[7] (Text 3, p. 1)

This perturbation is the intervention performed on the system aiming at revealing its internal causal structure. As a consequence, these interventions under the form of perturbations are the tools used for computing central magnitudes, such as the transition probability matrix and the cause repertoire of a mechanism:

The TPM is determined by the mechanisms of a system and obtained by perturbing the system into all its possible states.

[7] (p. 4)

The cause repertoire *p*(*ABC^p^*|*A^c^* = 1) is obtained via Bayes rule by perturbing the set of elements into all its states with equal likelihood.

[7] (Text 2, p. 1)

All the above-quoted claims suggesting a manipulabilist view of causation belong to the paper where the IIT 3.0 version was first presented [7]. Nevertheless, this paper does not explicitly refer to the work of Judea Pearl [52], according to which interventions (formally introduced in his theory by means of the *do(x)* operator) are used to identify causal relationships within interacting systems. Shortly after, in a 2016 paper, Tononi and his collaborators did acknowledge that their procedure

“is akin to the calculus of interventions and the *do(x)* operator introduced by Pearl (2000), to identify causal relationships”.

[55] (p. 2)

This first acknowledgment was immediately followed by a full development of a formal framework designed to provide a complete account of causation based on the manipulation of systems:

Given a causal network that represents how the state of each variable depends on other system variables via a ‘structural equation’ (Pearl, 2000), the effects of interventions imposed from outside the network can be evaluated by setting certain variables to a specific value. This operation has been formalized by Pearl, who introduced the ‘do-operator’, *do*(*X* = *x*), which signifies that a subset of system variables *X* has been actively set into state X rather than being passively observed in this state.

[56] (p. 2)

Up to this point, it is clear that the proponents of IIT and ourselves have reached the same conclusions by different paths. Whereas Tononi and his collaborators found in manipulabilism a theory of causation that was in resonance with their previous views in the context of the problem of consciousness, we arrived at manipulabilism in seeking for some conceptual means to design an interpretation of information free from the difficulties faced by the traditional interpretations. Now the question is about the specific relationship that links causation and information.

## 7. Information and Causation in IIT

Information and causation appear strongly linked in IIT from its first versions [3,4,5] to the most recent, where the interventionist version of the manipulabilist view of causation is explicitly introduced [36,55]. The two concepts have also played a relevant role in other proposals which, although related to IIT, aim to address more general problems. In this sense, it is worth mentioning an interesting contribution to the problem of emergence: by using precise tools coming from information theory, Tononi and his collaborators have developed convincing arguments directed toward showing that high-level causal relations may be stronger than those in the corresponding basal level [55,57] (This proposal introduces formal elements in the debate about emergence, usually discussed in terms not sufficiently precise. Thanks to one of the reviewers for emphasizing this aspect in the work of the proponents of IIT.) Nevertheless, it is still conceptually relevant to ask for the specific relationship between them: Is information defined in terms of causation? Is causation defined in terms of information? Are the two concepts equivalent?

In a very recent paper, Matthew Baxendale and Garrett Mindt [58] also propose an interventionist causal framework for IIT (We thank one of the reviewers for pointing out the need for considering Baxendale and Mindt’s paper.) in order to solve the so called “causal exclusion problem”, according to which mental properties cannot be part of causal relations due to their physical supervenience bases [59]. According to them,

[t]he informational account of causation they (the proponents of IIT) adopt renders their response damagingly circular, chiefly due to the account’s claim that the causal properties of a system are identical with its informational properties.

[58] (p. 331)

Are the advocates of IIT proposing an *informational account of causation*, which defines causation in terms of information? If this were the case, IIT would be aligned with those attempts to develop an informational theory of causation [53,60], according to which causation is nothing else than transference of information. This view, which tries to look into the very nature of causation, although philosophically consistent, requires supplying a satisfactory account of information. But, as explained in the previous section, this is not an easy task when scientific matters are taken into account. Moreover, if causation is defined in terms of information, the appeal to a manipulabilist view of information is forbidden at the risk of circularity: a manipulabilist causal view cannot be used to elucidate the concept of information by means of which causation will be defined.

Perhaps the strong link that IIT establishes between causation and information is due to the equivalence of the two concepts. This position is suggested by Tononi and his collaborators in claiming that:

IIT 3.0 explicitly treats integrated information and causation as one and the same thing.

[7] (p. 24)

Despite such an explicit statement, we think that this claim should not be taken literally. In fact, if the terms ‘causation’ and ‘information’ refer to one and the same thing, one of them is superfluous in the theory. It is not clear, then, why IIT is not called “integrated causation theory”, whose formalism supplies a way of quantifying causation.

The above remarks lead us to a clear conclusion. If IIT wants to retain a central role for both causation and information and, at the same time, to appeal to manipulabilist strategies, then the right approach consists in using manipulability to elucidate causation, and causation to elucidate information. To be clear, this is (i) to introduce the concept of causation in the definition of information, and (ii) to characterize causation in manipulabilist terms. The logical order would then be to use manipulability to elucidate causation, and causation to elucidate information. This is precisely the approach we propose with our manipulabilist interpretation of information.

Baxendale and Mindt [58] arrive at the same conclusion but from a different reasoning: they argue that the just described approach is necessary if IIT wants to solve the causal exclusion problem without falling into a circular argument. We have not appealed to the causal exclusion argument because it is one of the many arguments discussed in the field of the philosophy of mind, and it depends on several assumptions about inter-level causation that need to be previously assessed. Is it necessary that IIT solves this and other typical problems of the philosophy of mind to be an acceptable theory? This question leads us to the considerations of the next section.

## 8. IIT: Philosophical Proposal or Scientific Theory?

Although this question seems to lead us beyond the main aim of this paper, it needs to be considered to face certain criticisms against IIT related to its use of the concepts of causation and information.

Since the proponents of IIT repeatedly talk about the cause-effect power of mechanisms and systems, this might lead one to infer that they are committed to causal powers in a strongly ontological sense, as those discussed in the metaphysical debate on causal powers [61,62]. (We are grateful to one of the reviewers for calling our attention to the discussions about causal powers.) This view might be motivated by the fact that, in a recent paper, Tononi explicitly focuses on ontological concerns [63]. Nevertheless, the main point of his paper is not to advocate for the existence of metaphysical “causal powers” as ontic dispositions of things, but to appeal to the capability of an entity to take part of causal links as a criterion of its own existence. In fact, he is interested in “the requirements for something to exist and, furthermore, to exist for itself, as an intrinsic entity” [63] (p. 622), and he claims that “something can be said to exist only if it has cause-effect power” [63] (p. 622). But he conceives the cause-effect power, not as a metaphysical power intrinsic of things, but:

as established through observations and manipulations consistent with our best explanation of the regularities of experience itself.

[63] (p. 621)

This is the expected perspective of a scientist as Tononi, who, independently of his inner metaphysical beliefs, will argue in favor of his theory on empirical bases. Consistently, manipulabilism is a framework that explicitly claims its metaphysical neutrality (see [64]). As we asserted in Section 5, the interventionist version of manipulabilism supplies an *operational* criterion for causation, which is what empirical sciences need for fulfilling their goals.

Expecting the proponents of IIT to adopt a clear, well-based metaphysical stance has been a common tendency in the criticisms against IIT that treat it as if it were a philosophical proposal instead of a scientific theory. The main claim within this tendency is that IIT does not solve the hard problem of consciousness (see, e.g., [8,65,66]). Just to take one example, let us consider a recent paper where Mindt [67] concludes that IIT “leaves open the question of *why* experience is the result of integrated information, and so leaves open the hard problem of consciousness” [67] (p. 144). The author’s argument is based on requiring that physical properties *explain* consciousness, where the explanation is conceived in the logical terms of *derivation*:

But if one is not able to […] start with the physical system postulates and derive the phenomenological aspects of experience, then something is terribly amiss.

[67] (p. 152)

This shows that Mindt considers that the right explanation of conscious experience obtains only if consciousness is reduced, in Nagelian sense [68], to a physical basis. His reductionist demand is confirmed by his appeal to the case of water being H_2_O as a model of reduction: according to Mindt, the microstructuralist explanation in terms of structure and function of molecules of H_2_O is an exhaustive explanation of water, which tells us exactly why every instance of water is H_2_O, and conversely why every instance of H_2_O is water; such explanations of water and H_2_O also tell us at what temperature water/H_2_O reaches a boiling point, at which point it freezes, what particular conditions must obtain for it to go through state changes, etc.

The argumentative path followed by Mindt is illustrative because it plainly shows that he criticizes IIT not from the perspective of contemporary science, but on the basis of the assumptions of an outdated logical-empiricist philosophy of science. Chemists perfectly know that the exact boiling point of water cannot be strictly deduced from its microstructure (see [69]). Furthermore, philosophers of chemistry even claim that water is not strictly H_2_O, since it is composed of many different kinds of species involved in dynamical processes [70]. In general, the properties that chemists use to determine the sameness and purity of substances are macroscopic, such as boiling points, specific heats, latent heats, and so on. And from them much of the microstructure is actually inferred. Similarly, against the dreams of the logical-empiricist philosophers, thermodynamics and his concepts, such as temperature, have not been fully reduced to statistical classical mechanics (see [71]). Nevertheless, there is no “hard problem of temperature”, and physicists confidently operate with thermodynamics on the basis of its high empirical success. The complete derivation of consciousness from its physical basis may be an admissible desideratum in the context of the philosophy of mind, but is not a legitimate requirement for a scientific theory as IIT.

Another criticism of IIT that clearly shows a philosophical bias is Cerullo’s [66], who explicitly says that he examines the arguments of IIT from the viewpoint of the philosophy of mind. From this perspective he stresses, for instance, that a part of IIT’s axiom of information “is not a part of phenomenology”, where “we take phenomenology to include both its use in analytical philosophy and the continental school of phenomenology” [66] (p. 4), and that IIT does not fit easily into the traditional classification of—philosophical—theories of consciousness [66] (p. 5). As a strong argument against IIT, Cerullo proposes a trivial theory that he calls Circular Coordinated Message Theory (CCMT), whose justification “is the self-evident property that consciousness is related to information traveling in feedback loops within a system (the principle of information circulation)” [66] (p. 4). According to Cerullo, the fact that both theories, IIT and CCMT, have the same prediction and the same explanatory power in the case of split-brain syndrome “challenges the explanatory power of IIT” [66] (p. 4). The fact that two theories have the same explanatory power may be an obstacle to the search for the metaphysical nature of consciousness, but it is not a problem in science, where the Duhem–Quine thesis of the underdetermination of scientific theory by evidence [72,73] makes it logically possible for two different theories to have exactly the same predictions. Many philosophers of science have pointed out the difficulty of finding serious examples of underdetermination in real science; nevertheless, everyone recognizes that the Duhem–Quine thesis can always be exemplified by creating *trivial* theories that are empirically equivalent to successful scientific theories, and this is precisely what Cerullo does with his example. Circular Coordinated Message Theory would be a serious rival of IIT if it were developed enough to supply not only qualitative but also quantitative predictions, not only in one case but also in many different cases. But even if CCMT reached these scientifically desirable goals, this fact would not challenge the explanatory power of IIT; it would be an interesting case of actual underdetermination of the scientific theories about consciousness by the empirical evidence.

## 9. Conclusions

Integrated Information Theory is currently the leading theory of consciousness. Even though it was subject to several criticisms, many of them were successfully overcome in its most recent version, IIT 3.0. Some authors still point to certain limitations of the theory, such as the fact that it would only establish necessary conditions for consciousness and that it does not solve the hard problem of consciousness. But even if those or other limitations would be pertinent if IIT were conceived as a philosophical theory about consciousness, they do not threaten its scientific value: even the most reputed theories of physics have their limits of applicability. The fact that scientific theories have restricted boundaries, beyond which their results are no longer valid, is not a shortcoming of science, but an essential fact of scientific knowledge and the seed of its further development. In this sense, IIT has developed a scientific account of consciousness for more than a decade, improving its explanations and predictions in its subsequent versions. For this reason, it deserves to be seriously considered both from an empirical and from a conceptual viewpoint.

In this paper, we have taken a conceptual perspective devoted to analyzing the meaning of the word ‘information’ in the context of IIT. On the basis of our previous work on the interpretation of information and of the role played by the concept in the account of consciousness supplied by IIT, we have concluded that integrated information must be conceived from a causal–manipulabilist interpretation. According to this approach, information is supported by a structure of causal links, which are revealed by the results of manipulation. It is precisely this view of information that endows IIT with the power of supplying an objective and operationally effective account of consciousness with fruitful scientific content.

From a broader viewpoint, our paper aims at showing that the need for a precise elucidation of the concept of information is not specific to IIT: it is a generic requirement for any scientific theory that appeals to this concept, no matter how mathematically well-developed such a theory is. In this sense, our proposal places IIT into the broader framework of the scientific theories that make an essential use of the concept of information, and even suggests that, in spite of the differences among their formalisms, in all those theories, including IIT, the concept of information has the same core meaning strongly linked with manipulability.
